# HIV-1 infection induces a type I IFN response in primary CD4^+^ T cells

**DOI:** 10.1186/1742-4690-10-S1-P95

**Published:** 2013-09-19

**Authors:** Jolien Vermeire, Veronica Iannucci, Evelien Naessens, Kathleen Van Landeghem, Hanne Vanderstraeten, Jo Van Damme, Bruno Verhasselt

**Affiliations:** 1 Department of Clinical Chemistry, Microbiology, and Immunology, Ghent University, Gent, Belgium; 2 Laboratory of Molecular Immunology, Rega Institute, Faculty of Medicine, Katholieke Universiteit Leuven, Leuven, Belgium

## Background

Production of type 1 interferon (IFN) in response to viral infection requires the detection of viral nucleic acids or proteins by at least one cellular pattern recognition receptor. HIV-1 is capable of inducing an elaborate IFN response in plasmacytoid dendritic cells (pDCs) through Toll-like receptor mediated recognition of the entering viral RNA genome. However in T cells and macrophages, the main HIV target cells, IFN induction by HIV-1 is considered to be weak or undetectable. Here, we re-evaluate the occurrence of an innate immune response upon HIV-1 infection of primary CD4+ T cells (PBLs).

## Methods and results

Type 1 IFN induction was evaluated in activated purified PBLs during productive infection with HIV-1. In cells from several donors we observed a clear increase in IFNβ and IFNα mRNA levels, as well as induction of several interferon stimulated genes (ISGs) upon HIV-1 infection. The levels of induction progressed concurrently with the levels of HIV infection in the culture. Secreted type 1 IFN protein biological activity was furthermore detected in supernatants of HIV-1 infected cultures. To rule out residual contaminating pDCs as the source of IFN production in these cultures, we performed additional depletion of these cells from CD4+ T cell populations prior to infection and found that type 1 IFN induction was maintained in the absence of pDCs. Furthermore, we evaluated the biological relevance of the detected levels of type 1 IFN by addition of neutralizing antibodies to IFNβ and IFNα during infection. In presence of the both antibodies, we observed an increase in HIV-1 infection, indicating that the levels of HIV-induced IFN in the T cell cultures are sufficient to have an antiviral effect. To gain more insight in the mechanism of IFN induction by HIV-1, we used inhibitors of reverse transcription, integration and of the HIV-1 protease during single-cycle infection of VSV-pseudotyped HIV-1. We found that integration of the HIV provirus is required for full IFN induction in PBLs, indicating that newly expressed HIV RNA or newly produced HIV proteins are important for evoking an innate immune response in T cells.

## Conclusion

These data show that activated PBLs are capable of producing relevant levels of type 1 IFN in response to HIV-1 infection and suggest recognition of newly expressed HIV RNA or proteins as a main trigger of the innate response in these cells. Characterization of the responsible innate immune pathway and pattern recognition receptors will be subject of further investigation.

